# Bilateral Stafne Bone Cavity in the Body of the Mandible: An Unusual Case Report and Literature Review

**DOI:** 10.7759/cureus.39091

**Published:** 2023-05-16

**Authors:** Mayank Pahadia, Rutvi Vyas

**Affiliations:** 1 Oral and Maxillofacial Diagnostic Sciences, University of Florida, Gainesville, USA; 2 Oral and Maxillofacial Radiology, University of Florida Health, Gainesville, USA

**Keywords:** anatomical variation, three-dimensional imaging, cone-beam computed tomography (cbct), incidental radiological findings, bilateral lingual salivary gland depression, bilateral stafne bone cavity, bilateral stafne defect

## Abstract

Stafne defect also known as Stafne bone cavity is a lingual surface depression generally found in the posterior mandible. This entity is usually unilateral and asymptomatic, found during routine dental radiographic evaluation. Stafne defect appears as a well-defined, oval, corticated entity located below the inferior alveolar canal. These entities are the inclusion of the salivary gland tissues. In this case report, we present the case of a bilateral Stafne defect that was asymmetrically located in the mandible and was incidentally detected on the cone-beam computed tomography scan taken for implant treatment planning. This case report highlights the significance of three-dimensional imaging and correctly diagnosing the incidental findings within the scan.

## Introduction

Stafne defect or Stafne bone cavity (SBC) is classically described as an asymptomatic, unilateral depression on the lingual surface of the posterior mandible located between the inferior alveolar canal and the inferior border of the mandible. Several variations and atypical locations have been reported. In this case report, we present one such case of a bilateral Stafne defect which was asymmetric and presented in atypical locations and highlight the importance of imaging in the diagnosis of this entity. In 1942, Edward Stafne described an entity found unilaterally in the posterior region of the mandible, which was a radiolucent cavity between the mandibular angle and the third molar and below the inferior alveolar canal and referred to it as SBC [1]. Since then, multiple researchers have described this entity, and now it is known under several synonyms, namely, SBC, lingual salivary gland depression, lingual mandibular bone depression, developmental salivary gland defect, Stafne defect, static bone cavity, and idiopathic bone cavity [2]. Aps et al. analyzed 64 papers discussing this entity and found that these lesions can occur anywhere in the mandible and can occur unilaterally or bilaterally. Hence, they proposed to rename the entity as “benign mandibular concavity” [3]. The purpose of this case report is to present a rare case describing a Stafne defect occurring bilaterally in the posterior mandible found as an incidental finding on routine panoramic and cone-beam computed tomography (CBCT) imaging for dental implant planning.

## Case presentation

**Figure 1:**
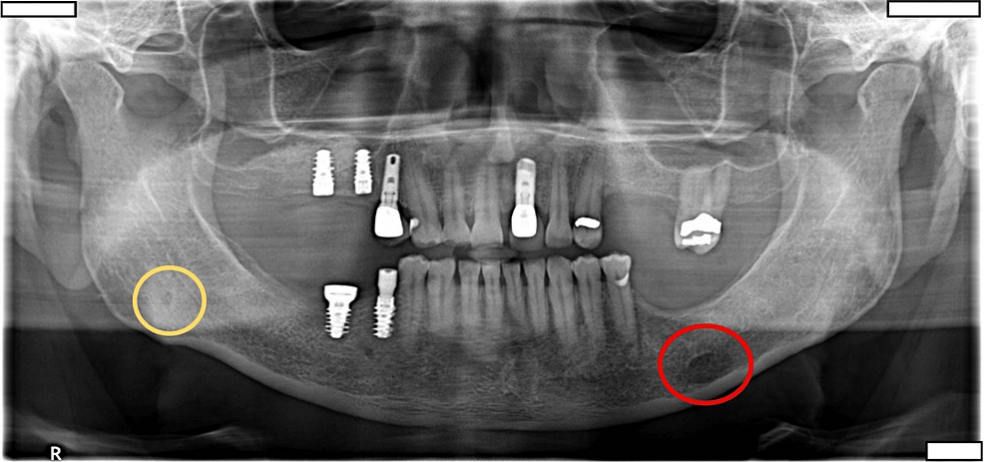
Panoramic radiograph showing well-defined radiolucency bilaterally in the body of the mandible encircled within the yellow and red circles.

**Figure 2:**
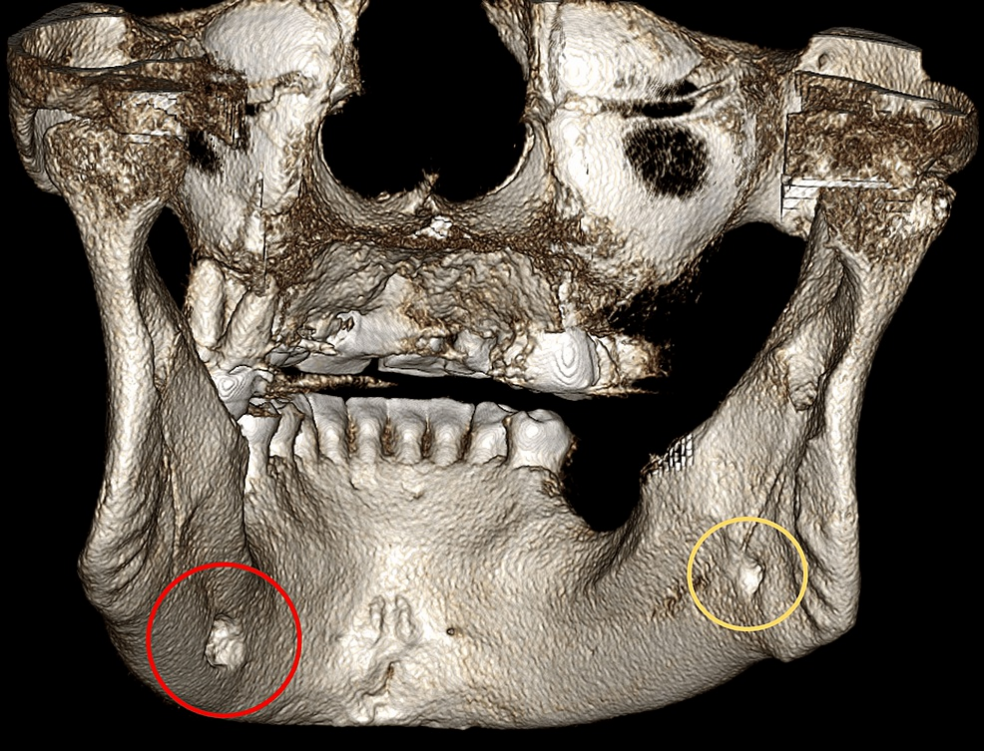
Volume-rendering reconstruction showing bilateral concavities on the lingual cortical surfaces. This is the posterior view of the mandible.

**Figure 3:**
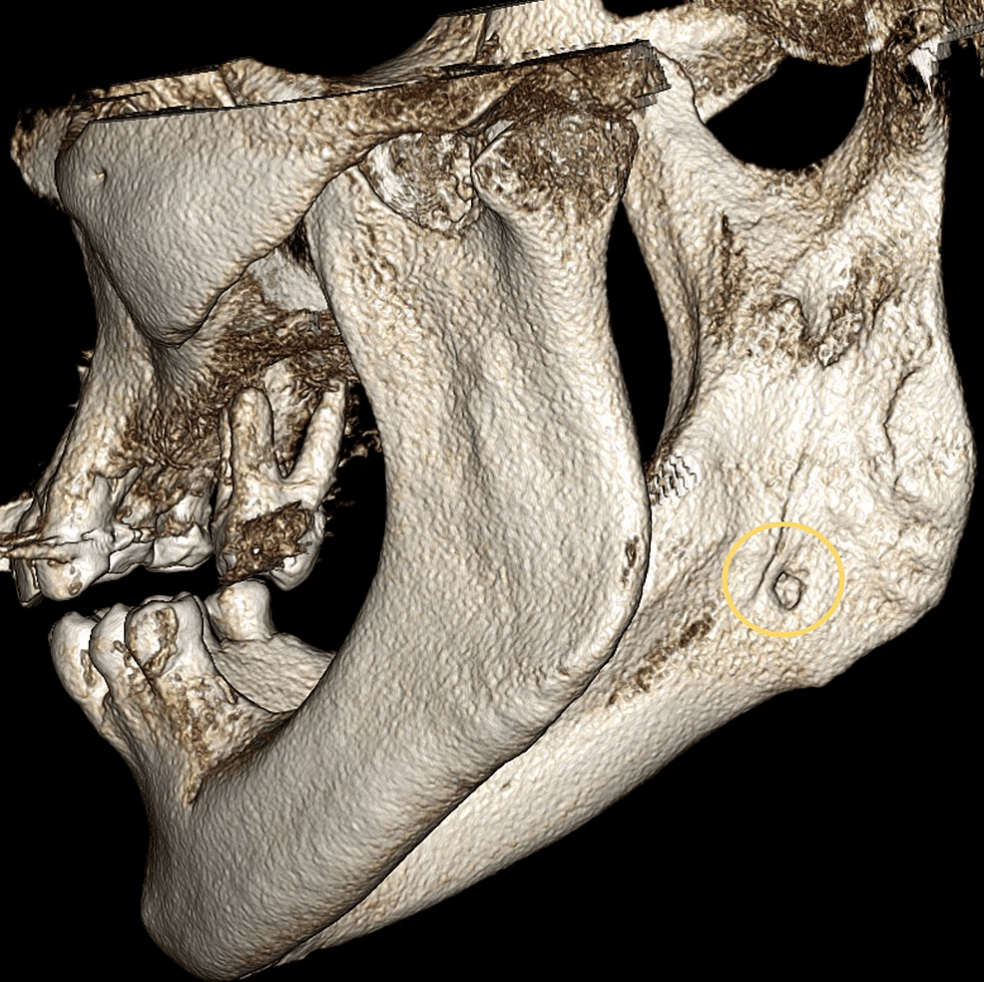
Volume-rendering reconstruction showing concavity on the right lingual cortical surface. This is the posterolateral view (right side) of the mandible.

**Figure 4:**
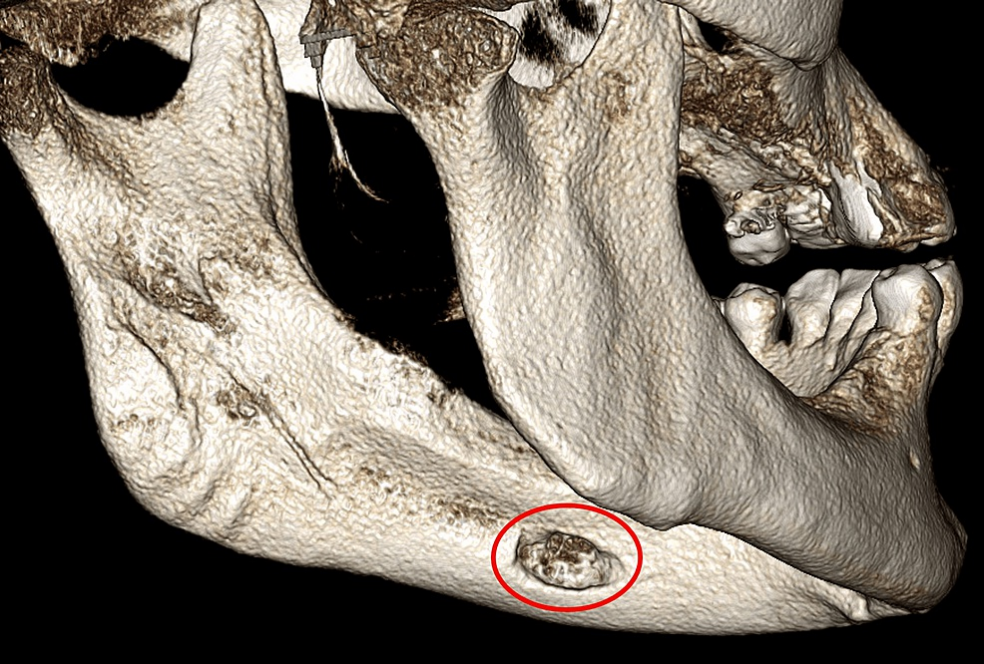
Volume-rendering reconstruction showing concavity on the left lingual cortical surface. This is the posterolateral view (left side) of the mandible.

**Figure 5:**
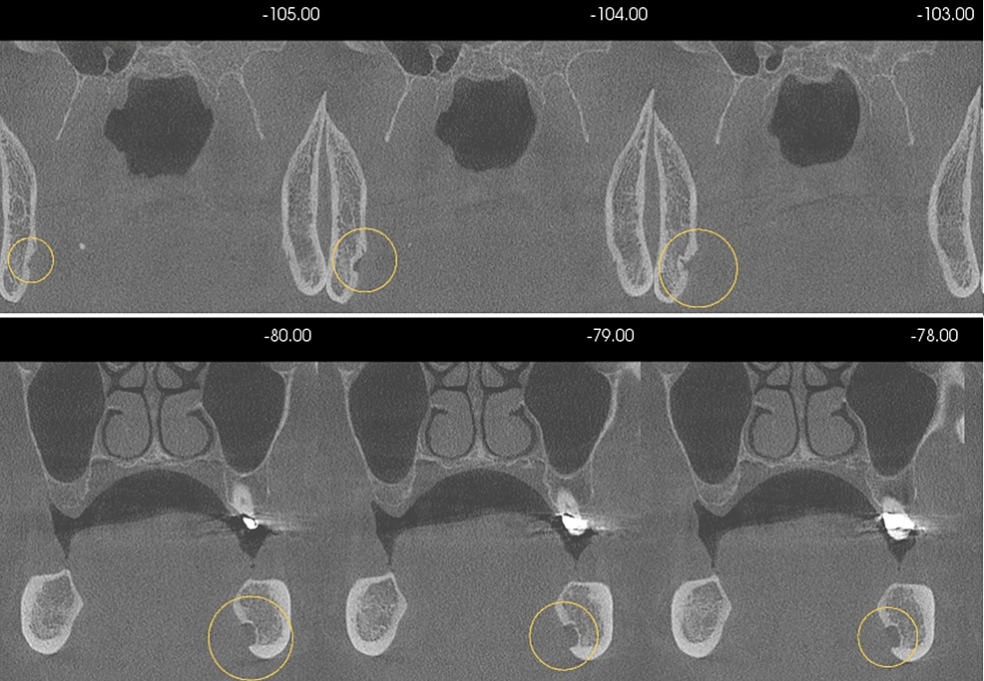
Multiple coronal cross-sections at 1.0 mm slice intervals showing bilateral lingual cavities with an intact lingual cortex.

**Figure 6:**
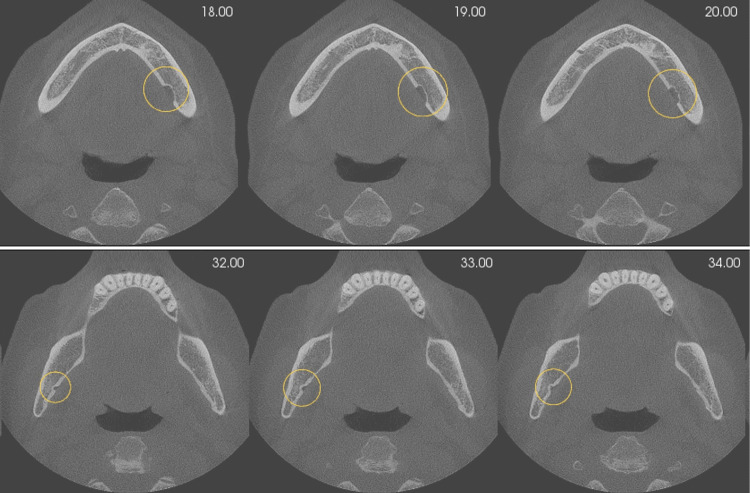
Multiple axial cross-sections at 1.0 mm slice intervals showing bilateral lingual cavities with an intact lingual cortex.

A 62-year-old male visited the University of Florida, College of Dentistry with a chief complaint of missing teeth in his upper and lower left jaw regions. He had no significant medical history and was completely asymptomatic. On routine panoramic radiographic examination, we found a well-defined, partly corticated radiolucency in the left mandible superimposed over the inferior alveolar canal at the level of the edentulous space of 19 (Figure 1). Another radiolucency, smaller in dimension and mostly non-corticated, was noted in the right angle of the mandible region inferior to the inferior alveolar canal (Figure 1). The preliminary radiographic diagnosis for the entity on the right side was a Stafne defect, while that on the left side needed to be further evaluated as it was an atypical location for Stafne. The differential diagnosis of that on the left side included Stafne defect, other entities such as an accessory mental foramen, or less likely a pathological entity. Subsequently, a medium field-of-view CBCT scan was acquired for evaluation of the bone for implant planning as well as to rule out a possible pathological entity about the radiolucency on the left side. The three-dimensional volumetric reconstruction showed an intact and smooth lingual cortical outline with a cavity along the surface (Figures 2-4). The multiplanar reconstruction of the CBCT study in axial and coronal planes showed a well-defined, lingual concavity measuring approximately 9.5 mm × 7.4 mm in the left body of the mandible at approximate site 19 and measuring approximately 2.4 mm × 4.7 mm in the right angle of the mandible. Both entities were located inferior to the respective inferior alveolar canals (Figures 5, 6). Based on these findings, a radiographic diagnosis of bilateral Stafne defect was made and an appropriate recommendation was provided to the referring dentist that no further diagnostic or therapeutic intervention was required.

## Discussion

The Stafne defect is a rare anomaly found as a depression along the lingual surface of the mandible. It is believed that the cavity could be developmental as the literature reports the inclusion of tissues of the submandibular salivary gland or even fatty tissues. These bone cavities are incidentally identified during routine dental evaluation as these patients are generally asymptomatic [4,5]. Although it is uncommon, it may rarely cause expansion of the jaw [6,7].

The literature has reported varied prevalence of SBC; however, most studies have reported a lower range of around 0.10% to 0.48%, with a higher incidence in the male population [5]. Although there is a wide variety of age ranges noted among the reported cases in the literature, the majority of them are within the age group of the fourth to sixth decade of life with no definitive race predominance [5,8].

The etiopathogenesis of SBC is not well studied and documented in the literature. However, several different theories have been proposed to understand the development of the cavity, which include the possibility of it being developmental in origin with failure of proper ossification versus salivary gland inclusion versus variation in anatomy at birth considering it congenital by nature [6].

The diagnosis of the Stafne defect is usually based on radiographic imaging. Routine dental imaging including panoramic radiograph(s) is usually enough for a preliminary radiographic diagnosis; however, Stafne defect can occur at atypical locations, including the anterior mandible [9], the ramus region [10], and can sometimes mimic other entities such as an accessory mental foramen [11], which is an anatomical variation, or, on rare occasions, a pathological entity, such as a benign cyst [9].

Very few cases with bilateral SBC are published in the literature, with only three other case reports reported so far according to a PubMed search. A study by Queiroz et al. reported a bilateral Stafne defect in the anterior mandibular region apical to canine and incisors [12]. Another study by Junquera et al. reported a case of bilateral SBC [13]. Boyle et al. also reported an unusual case of three SBCs, two in the posterior mandible and one in the para-symphyseal region [14]. Multiple Stafne defects need to be carefully evaluated using advanced imaging modalities such as CBCT to rule out multi-foci radiolucent lesions such as multiple myeloma. In these cases, CBCT imaging can provide optimal visualization and confirm the radiographic diagnosis of the Stafne defect. A biopsy is usually not indicated as a radiographic diagnosis is enough; however, there may be instances where an uncommon location or size of the Stafne defect may mimic other pathological entities, such as a benign cyst or tumor, and histopathological correlation would be required to confirm the diagnosis [7].

SBCs do not require any treatment. The most common management recommendation is observation. Periodic radiographic imaging with conventional modalities may be performed to monitor the size and shape of the defect over time [8].

## Conclusions

SBC can be present bilaterally with asymmetric sizes and at different locations; therefore, careful radiographic evaluation is imperative in reaching the correct diagnosis. Three-dimensional imaging modality can play a significant role in the differential diagnosis when multiple such defects are present, and when there is a need to rule out any other pathology in the area.
